# Determination of thermodynamic parameters of Xerocomus chrysenteron lectin interactions with N-acetylgalactosamine and Thomsen-Friedenreich antigen by isothermal titration calorimetry

**DOI:** 10.1186/1471-2091-6-11

**Published:** 2005-06-01

**Authors:** Luminita Damian, Didier Fournier, Mathias Winterhalter, Laurent Paquereau

**Affiliations:** 1 IPBS UMR 5089, Biotechnologie des Protéines, 205 route de Narbonne, 31077 Toulouse, France; 2 IUB, School of Engineering and Science, D-28727 Bremen, Germany

## Abstract

**Background:**

Lectins are carbohydrate-binding proteins which potentially bind to cell surface glycoconjugates. They are found in various organisms including fungi. A lectin from the mushroom *Xerocomus chrysenteron *(XCL) has been isolated recently. It shows insecticidal activity and has antiproliferative properties.

**Results:**

As the monosaccharide binding specificity is an important determinant of lectin function, we determined the affinity of XCL for the galactose moiety. Isothermal titration calorimetry studies revealed a dissociation constant K_d _of 5.2 μM for the XCL:N-acetylgalactosamine interaction at 27degreesC. Higher affinities were observed at lower temperatures and higher osmotic pressures. The dissociation constant was five hundred times higher for the disaccharide beta-D-Gal(1–3)-D-GalNAc, Thomsen-Friedenreich (TF) antigen (Kd of 0.94 μM). By using fetuin and asialofetuin in interaction with the XCL, we revealed its ability to recognize the Thomsen-Friedenreich motif on glycoproteins.

**Conclusion:**

The XCL antiproliferative effect and the TF antigen specificity presented in this work suggest that XCL and ABL may have similar binding mechanisms. The recent structure determination of these two proteins lead us to analyse these interactions in the light of our thermodynamic data. The understanding of this type of interaction may be a useful tool for the regulation of cell proliferation.

## Background

Lectins are carbohydrate-binding proteins found in various organisms including fungi [1,2]. Despite the large amount of informations available on lectin sequence and specificity, relatively little is known about their biological significances. The abundance and the variety of carbohydrate specificities of lectins raised the interest to use them for isolation and analysis of complex carbohydrates, cell separation and studies of cell surface architecture [3]. For a long period, legume lectins were the model system of choice to study the molecular basis of carbohydrate-lectin recognition. They are easy to purify in large quantities, and they exhibit a wide variety of carbohydrate specificities despite strong sequence conservation [4].

Mushroom lectins have captured the attention of investigators on account of their antiproliferative, immunomodulatory, antitumor and cytotoxic activities, and more than 50 mushroom lectins have been reported [5]. We recently isolated a lectin from *Xerocomus chrysenteron *(XCL) [6]. The X ray crystal structure resolution of XCL revealed a tetrameric assembly and an unexpectedly similarity with actinoporins [7]. XCL was reported as an insecticidal protein [6] and shares antiproliferative properties against two mammalian cell lines [8]. We can also mention *Agaricus bisporus *lectin (ABL), another mushroom lectin well known for its reversible antiproliferative effects [9].

ABL is a member of a group of proteins, which bind the Thomsen-Friedenreich (TF) antigen selectively and with high affinity. TF antigen is represented by galactosyl β-1, 3-N-acetylgalactosamine and is common in malignant and pre-malignant epithelia [10,11]. There are three other well known dietary TF-binding lectins: jacalin from the seeds of jackfruit *Artocarpus integrifolia*, the peanut lectin from peanut *Arachis hypogaea*, and amaranth lectin from *Amaranthus caudatus*. These four lectins have been used in histochemistry for identification of the TF antigen in tissues [12,13].

As previously reported by Rosen and al. [14], ABL belongs to a lectin fungi family based on sequence homology and N-acetylgalactosamine and galactose affinity. At present this family contains: *Agaricus bisporus *lectin (ABL), *Arthrobotrys oligospora *lectin, *Xerocomus chrysenteron *lectin (XCL), *Pleurotus cornucopiae *lectin, *Gibberella zeae *lectin, *Paxilus involutus *lectin [15]. The sequence homology between XCL and its family members varies from 65% to 35% suggesting that all these lectins could recognize TF antigen.

Here we focus on XCL binding constants for specific sugars and quantify the underlying thermodynamic parameters of the carbohydrate-XCL lectin interactions by direct measurement of the enthalpy using isothermal titration calorimetry method. We found that XCL recognizes TF antigen with high affinity (Kd: 1 μM).

## Results

### Sugar – XCL interaction

Red blood cell agglutination by XCL was inhibited when galactose, lactose and N-acetylgalactosamine was added to the system but no effect was seen with glucose, fucose, fructose, sorbitol, mannose and sucrose [6]. We first performed a titration of lactose and galactose to the protein, however the low binding affinity of both sugars was below the detection limit of the method (data not shown). Subsequently, we investigated the N-acetylgalactosamine/XCL interaction. With N-acetylgalactosamine, the acetamide group on the galactose ring can bring one more hydrogen bond, which can contribute to the enthalpy of the reaction and affinity values, and then titration was possible [16].

**Figure 1:**
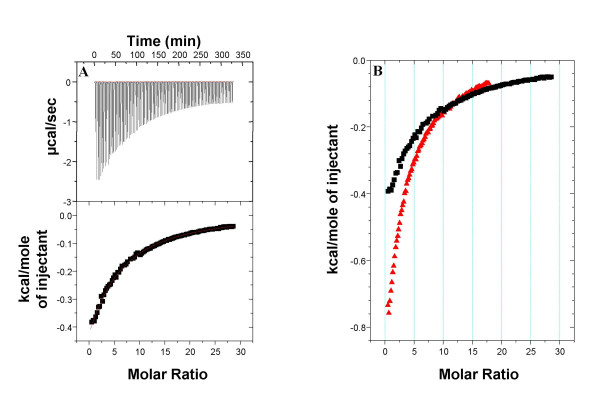
ITC profile and treatment of data of XCLtag and N-acetylgalactosamine interaction using 25 mM Na_2_HPO_4_/NaH_2_PO_4 _as buffer. A: Top: raw data obtained from 98 automatic injections (3 μl each), by titration of 50 mM N-acetylgalactosamine into 0,394 mM XCLtag solution. Bottom: the integrated curve showing the experimental points (■) and the best fit (-) B: Comparison of titrations realized at two different temperatures. Red scatter (): 10°C data titration; black scatter (■): 27°C data titration.

Figure 1A shows a titration of N-acetylgalactosamine into XCL protein at 27°C, together with a least squares fit. An apparent monotonic decrease in the heat release evolves when increasing amount of ligand is added, suggesting that XCL displays only one type of binding site and the absence of allostery between the four sites present on the tetramer. The fit of the data based on the one type site model reveals a binding constant of 192 M^-1 ^and a reaction enthalpy of – 6.27 kcal/mol when the monomer concentration was considered for the calculations. As this affinity is very small, the enthalpy was estimated separately in another experiment where small quantities of lectin were injected into a N-acetylgalactosamine containing solution. We obtained a ΔH value of – 25.20 kcal/mole corresponding to four independent binding sites (data not shown).

Binding of carbohydrates to a number of proteins is characterised by small enthalpy and heat capacity changes. Hydrogen bonding interactions are essentially enthalpically driven with little change in the heat capacity, while hydrophobic interactions are essentially entropically driven [17]. Measurements performed at 10°C using the same titration conditions indicate that the enthalpy of binding of N-acetylgalactosamine does not vary significantly with temperature and small changes in the heat capacity are observed. The fit of the data with one set of site model (figure 1B, red spectra) gave an affinity value of 362 M^-1 ^and no important change of the reaction enthalpy was observed (- 6.25 kcal/mole). In many cases, binding of saccharides to lectins is coupled to changes in solvent accessibility that result in negative, albeit small, ΔCp values [16]. This is also the case of XCL – N-acetylgalactosamine interaction.

Variation of osmotic stress allows to measure the energetic contribution of the solvatation effect on the enthalpy of the reaction [18]. The water activity was reduced by adding 10 % (w/v) PEG 8000 to the system. An increase in the binding constant value (280 M^-1^) and a reduction of the binding enthalpy (- 5.82 kcal/mole) were observed. Thermodynamics parameters, which characterize the XCL – N-acetylgalactosamine interaction are summarised in Table 1. The ΔG values and the deduced binding constants are higher at low temperature or under osmotic stress.

**Table 1:** Thermodynamic values, which characterize the interaction of XCL with N-acetylgalactosamine

	Ka, M^-1^	-ΔH, kcal/mole	-ΔG, kcal/mole	-ΔS, cal/moleK	Nr exp.
27°C	192 ± 5	6.27 ± 0.10	3.122	10.5	2
27°C, 10% PEG	280 ± 4	5.82 ± 0.05	3.347	8.2	4
10°C	362 ± 3	6.25 ± 0.03	3.301	10.4	2

The N-acetyllactosamine was also used as a ligand and the affinity constant at 27°C is in the range of 50 M^-1 ^but with significant errors. These errors are due to an uncertainty in fitting the data at Ka values of smaller than 100 M^-1^.

**Figure 2:**
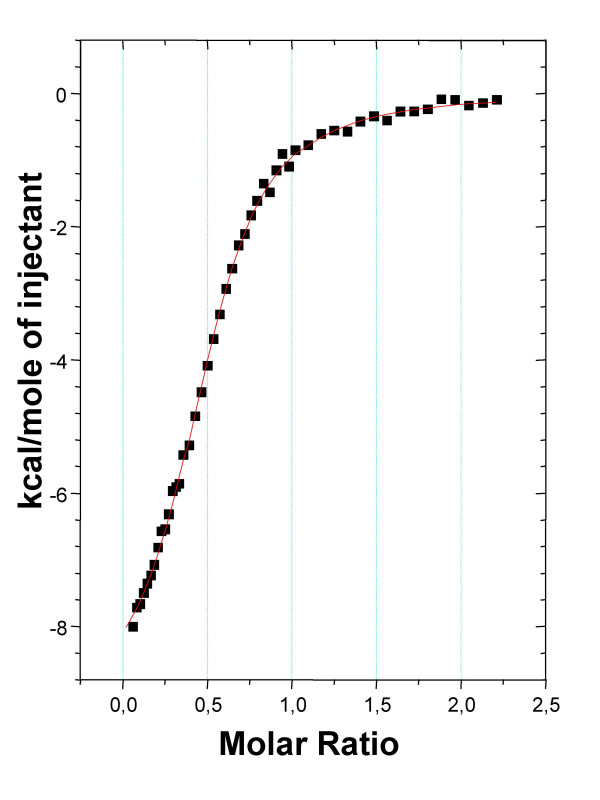
Binding isotherms acquired by titration of 0.95 mM β-D-Gal(1→3)-D-GalNAc into 0,14 mM XCLtag solution at 27°C, using 25 mM Na_2_HPO_4_/NaH_2_PO_4 _as buffer.

We especially checked the XCL interaction for TF antigen, β-D-Gal(1–3)-D-GalNAc, since ABL was previously shown to bind this disaccharide with a high affinity constant. A ΔH value of -9.13 kcal/mole and an affinity constant of 1.06 10^5 ^M^-1 ^were obtained (fig. 2 and table 2). This value is 500 fold higher than the affinity constant determined for N-acetylgalactosamine interaction.

**Table 2:** Thermodynamic values of binding with Thomsen-Friedenreich antigen (TF), fetuin and asialofetuin

	Ka, 10^5 ^M^-1^	-ΔH, kcal/mole	-ΔG, kcal/mole	-ΔS, cal/moleK	Nr exp.
TF	1.06 ± 0.44	9.13 ± 0.29	6.81 ± 0.27	7.61	2
Fetuin	5.9 ± 1.4	21.5 ± 0.5	7.89	45.35	3
Asialofetuin	25.9 ± 0.6	16.8 ± 2.5	8.84	26.51	2

### Glycoproteins – XCL interaction

Fetuin and asialofetuin, which bear the TF antigen motif, were used to test XCL interactions with glycoproteins. Fetuin contains six oligosaccharides chains, namely three carbohydrate units O-linked to Thr or Ser residues and three complex glycans, N-linked to Asn residues [19]. A fourth O-linked residue may exist in the fetuin structure [20]. In fetuin, the exposed Gal residues of both O-linked and N-linked saccharides are linked to sialic acid residues, which are absent in asialofetuin.

**Figure 3:**
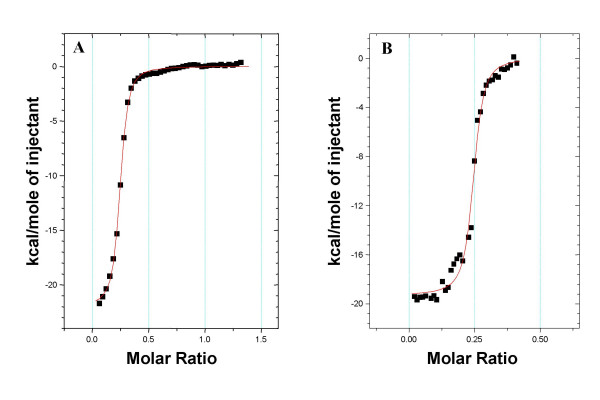
Binding isotherms corresponding to the titration of 38 automatic injections (5 μl each) of 2 mM fetuin (A) or 2 mM asialofetuin (B) into 0,25 mM XCLtag solution at 27°C, using 25 mM Na_2_HPO_4_/NaH_2_PO_4 _as buffer.

Several titrations of fetuin and asialofetuin to a XCL containing solution (see concentrations in material and methods) were performed at 27°C. The binding isotherms for the titration of fetuin and asialofetuin into a XCL solution are presented in figure 3A and 3B respectively and the thermodynamic data are presented in Table 2. Affinity constant for asialofetuin (2.59 10^6 ^M^-1^) was found 4 times higher than for fetuin (5.9 10^5 ^M^-1^). This suggests that XCL binds asialofetuin more avidly than the native fetuin, and therefore that the presence of sialic acid reduces the affinity of XCL towards such glycans. The binding stoichiometry is of 0.23, which could correspond to 4 similar binding sites either on fetuin or asialofetuin. The binding enthalpy of XCL – fetuin/asialofetuin is of -21.5 kcal/mole and -16.8 kcal/mole respectively. This significant difference in the binding enthalpies of almost 5 kcal/mole leads us to conclude that in fetuin the sialic acids do contribute to the energy of binding.

## Discussion

As we mentioned in the introduction, there are several TF-binding lectins. Althought they recognize the same motif, they have different actions on the proliferation phenomenon [21]. For example, PNA stimulates the proliferation of human intestinal epithelial cells [22] while ABL is a potent inhibitor of proliferation [9]. The fact that XCL shows a dose-dependent inhibition of proliferation [8] suggests that its effects could be mediated by glycoproteins bearing TF antigen. We first check the binding of XCL with free TF antigen. Our results lead us to suggest that water molecules involved in the sugar-lectin binding may contribute to the energy of the reaction. This is in agreement with the Chevernak and Toone work since the amount of heat liberated on the binding of ligands with a variety of proteins was significantly smaller (0.4 – 1.8 kcal/mol) when heavy water was used like solvent [18]. In the case of XCL, the affinity enhancement observed when the galactose is linked to the N-acetylgalactosamine suggests the existence of an extended binding site [23]. An increase in the binding enthalpy is also observed when disaccharides replace monosaccharides in XCL-sugar complexes. This increase correlates with the addition of direct hydrogen bonds and more extensive van der Waals [24] interactions between the protein and the ligand. Sugar binding site determined on ABL by RX crystallography shows that water molecules are involved in this interaction as we hypothesised [25].

On cell-surface glycoproteins, the epitope structure of TF antigen is α-linked to either serines or threonines [26]. The affinity constants of XCL obtained for fetuin and asialofetuin are higher than for free TF antigen. This difference could be explained by an implication of several residues of the glycoprotein in the interaction with the lectin. Nevertheless, residues potentially involved in this interaction are not serine or threonine linking TF antigen [25]. Then it would be interesting to investigate the implication of the spatially surrounding residues in this interaction.

## Conclusion

At present, only limited informations on the thermodynamics datas of the lectin-sugar recognition are available and much work remains to be done to understand the underlying forces that govern these interactions. In this study, we investigate the specificity of XCL for carbohydrates and especially for Thomsen-Friedenreich antigen and glycoproteins bearing this disaccharide. Kinetic studies using a resonant mirror biosensor reported a binding affinity value of 3.3 10^6 ^M^-1 ^for the asialofetuin-ABL interaction [27] which is very close to that of the asialofetuin-XCL interaction (2.59 10^6^M^-1^). The XCL antiproliferative effect [8] and the TF antigen specificity presented in this work suggest that XCL and ABL may have similar binding mechanisms. The recent structure determination of XCL and ABL lead us to currently analyse these interactions in the light of our thermodynamic data.

## Methods

### Materials

All products, mono- and di-saccharides, fetuin and asialofetuin from fetal bovine serum were purchased from sigma.

### XCL expression and purification

A fusion protein containing histidine tag, TEV site and XCL was expressed in *E. coli *BL21-DE3 strain. The histidine tag was added to facilitate the purification of the recombinant protein on an affinity column using nickel as ligand [28] and the TEV site was added to eliminate the tag by incubation with TEV protease [29]. Freshly transformed BL21(DE3) cells were grown overnight in a NZY/agar – kanamycin medium at 37°C. Colonies of bacteria were grown in an NZY medium at 37°C. When an O.D._600 nm _of 1 was reached, the induction of T7 RNA polymerase with IPTG (final concentration 0.4 mM) was realized. Then the culture medium was allowed to grow overnight, at 16°C. Cells were harvested by centrifugation, washed and then lysed by sonication. Isolated XCL was purified by affinity chromatography on Ni-NTA column and dialyse methods. Protein purity was assessed using overloaded SDS-PAGE gels with Coomassie blue staining. XCL concentrations were determined spectrophotometrically from molar extinction coefficients at λ = 280 nm, ε = 31150.

### Isothermal titration calorimetry, ITC

Isothermal titration calorimetry was performed using a VP-ITC microcalorimeter from Microcal Inc. (Northampton, MA). Several experiments were performed to determine the binding constant values. In individual titrations, injections of 3 to 10 μl of carbohydrate/glycoprotein were added by computer-controlled 296 μl microsyringe at an interval of 200 seconds into the XCL solution (cell volume = 1.437 ml). The experiments were realized at 27°/10°C and a stirring speed of 300 rmp. 10% (w/v) PEG 8000 was used for some of the experiments. As the lectin affinity for sugars is relatively small, high sugar and protein concentrations were required. The XCL concentration varied between 0.14-0.4 mM, the monosaccharide concentrations between 30–50 mM, 0.95–3 mM for TF antigen and glycoproteins concentrations between 0.66–2 mM. The carbohydrates were dissolved in the buffer solution (25 mM Na_2_HPO_4_/NaH_2_PO_4_, pH = 7) from the last protein dialysis.

Several blind titrations were performed to determine and correct for unspecific heat contributions (heat of dilution).

The experimental data were fitted to a theoretical titration curve using software supplied by Microcal, with ΔH (enthalpy change in kcal/mole), K_a _(association constant in M^-1^) and n (number of binding sites), as adjustable parameters. The monomer concentration was used throughout the analysis. The instrument was calibrated using the built in mode of electric field heat pulses. Thermodynamic parameters were calculated from the relation:

Δ*G *= Δ*H *- *T*Δ*S *= - *RT *ln *K*_*a*_

where the ΔG, ΔH and ΔS are the changes in free energy, enthalpy and entropy of binding; T is the absolute temperature and R = 1.98 cal mol^-1^K^-1^.

## Authors' contributions

LD carried out the protein preparation and microcalorimetry work and drafted the manuscript. DF participated in the design of the study, MW participated in the microcalorimetry experiment and interpretations. LP conceived of the study, and participated in its design and coordination. All authors read and approved the final manuscript.
